# Avoiding invasive surgical procedures for a slow‐growing asymptomatic huge anterior mediastinal lipoma under watchful waiting

**DOI:** 10.1002/ccr3.5190

**Published:** 2021-12-18

**Authors:** So Motomura, Shun Yamashita, Masaki Tago, Shu‐ichi Yamashita

**Affiliations:** ^1^ Department of General Medicine Saga University Hospital Saga Japan; ^2^ Department of General Medicine Yuai‐Kai Foundation & Oda Hospital Kashima Japan

**Keywords:** anterior mediastinal lipoma, asymptomatic mediastinal tumor, intrathoracic lipoma, thoracic huge mass lesion, watchful waiting

## Abstract

A mass shadow in the right lower lung field was detected by chest X‐ray in a 79‐year‐old woman, which was eventually diagnosed as anterior mediastinal lipoma. She remained under watchful waiting without surgery for 9 years without developing symptoms, even though the lipoma had grown to an extremely large size.

## Graphical abstract

Unlike symptomatic mediastinal lipomas causing respiratory symptoms or heart failure, we might be able to avoid invasive and burdensome surgical resection to treat an asymptomatic mediastinal lipoma, even with extremely large size, only by watchful waiting.

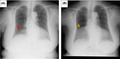

## 1 CASE PRESENTATION

**FIGURE 1:**
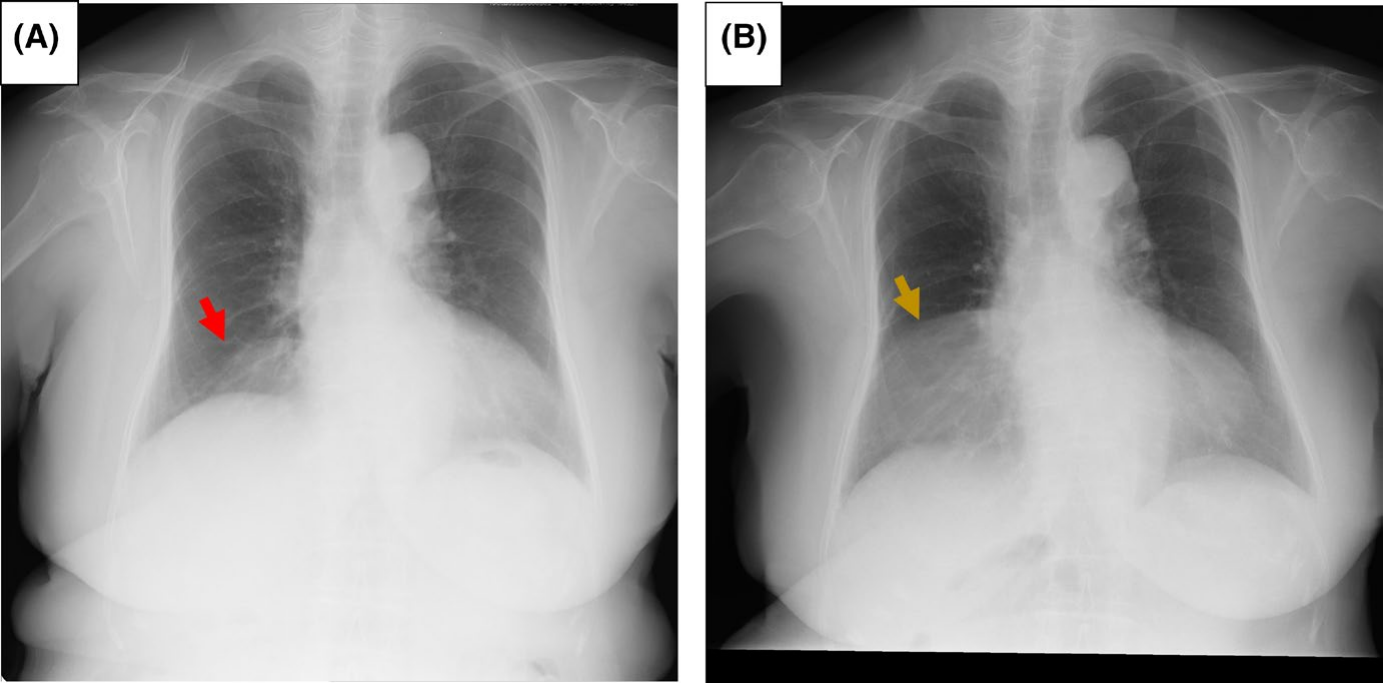
Findings of the chest X‐ray. (A) on her first visit, (B) nine years later. Chest X‐ray showed a mass shadow in the right lower lung field (red arrow). The mass shadow has significantly grown in the past 9 years (yellow arrow)

**FIGURE 2:**
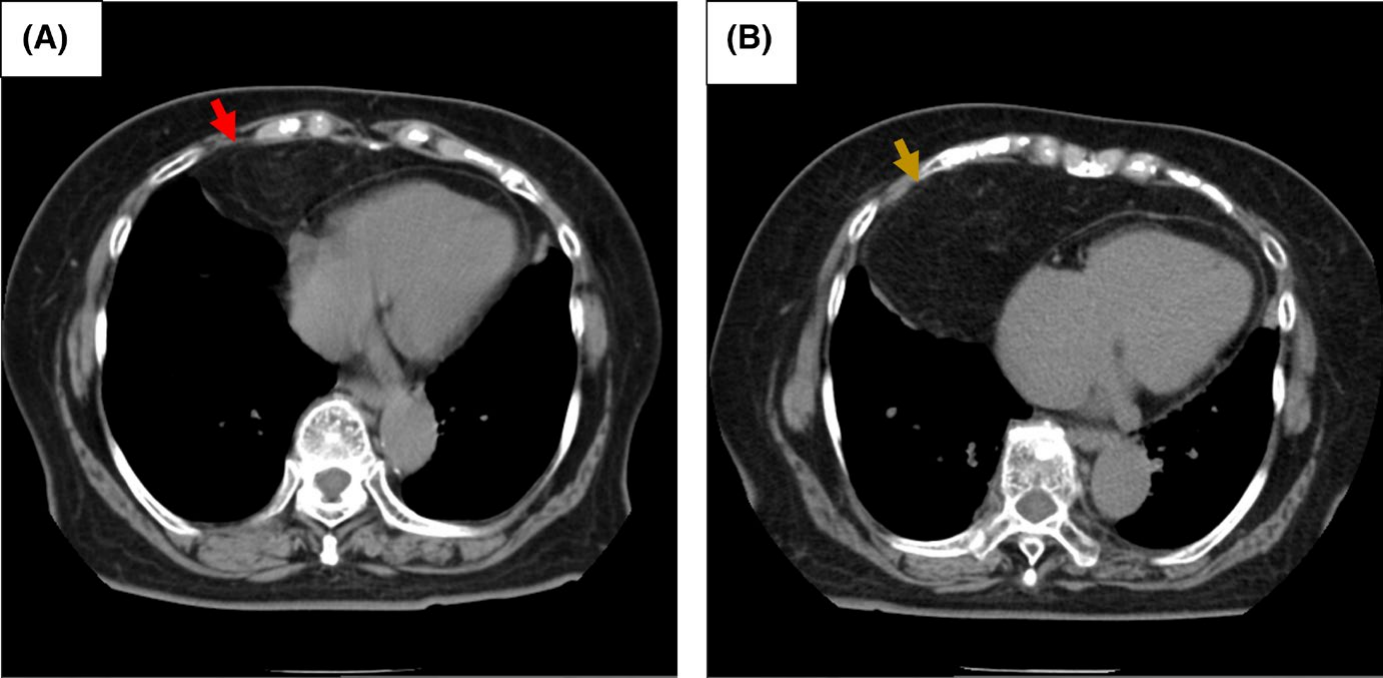
Findings of the thoracic CT without enhancement. (A) on her first visit, (B) nine years later. Thoracic CT showed a homogenous mass shadow measuring 17.4 cm × 4.5 cm × 6 cm in her right anterior mediastinum (red arrow). The mass shadow has significantly increased in the size of the mass to 18.9 cm × 8.0 cm × 8.0 cm in the past 9 years (yellow arrow)

**FIGURE 3:**
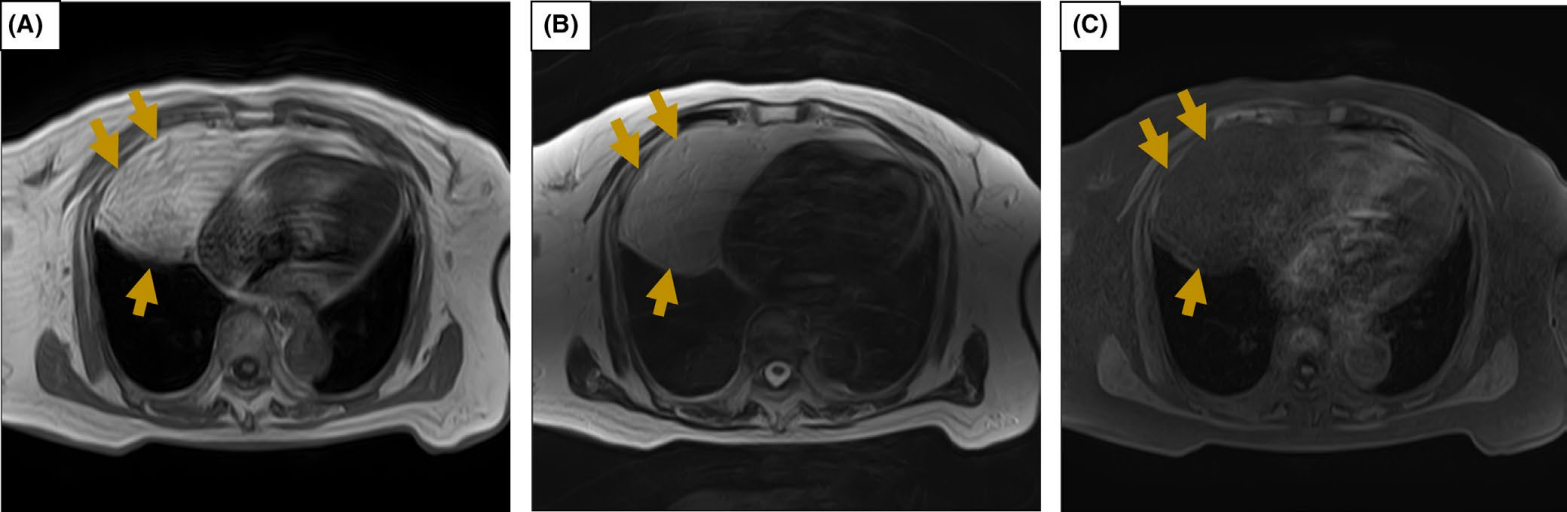
Findings of the recent MRI. (A) T1‐weighted image, (B) T2‐weighted image, (C) Fat suppression technique image. MRI showed a homogenous mass with smooth edge, with the signal intensity reduced by the fat suppression technique (yellow arrow). These findings suggested the diagnosis of mediastinal lipoma without component of liposarcoma

A 79‐year‐old woman underwent chest X‐ray, which revealed a mass shadow in the right lower lung field (Figure 1). Thoracic computed tomography (CT) revealed a homogenous (−100 Hounsfield units) mass lesion measuring 17.4 cm × 4.5 cm × 6 cm in her right anterior mediastinum (Figure 2A). Being asymptomatic, she was managed under watchful waiting for 9 years. Follow‐up recent CT showed that the mass lesion increased to 18.9 cm × 8.0 cm × 8.0 cm (Figure 2B). T1‐ and T2‐weighted images on magnetic reasoning image showed a homogenous mass with smooth edge, with the signal intensity reduced by the fat suppression technique (Figure 3).

## 2 DISCUSSION

Mediastinal lipoma is a type of intrathoracic lipoma. Although a symptomatic intrathoracic lipoma requires removal,^1^, ^2^ surgery for an asymptomatic intrathoracic lipoma is controversial. Surgical resection of huge intrathoracic lipomas could injure adjacent structures, such as nerves or vessels. Furthermore, there are reports of asymptomatic intrathoracic lipomas successfully managed by a conservative approach.^2^ Specifically, an asymptomatic mediastinal lipoma, even when extremely large, could be managed under watchful waiting, as in our patient, who remained asymptomatic for 9 years, until the age of 88 years, with unimpaired activities of daily living.

## CONFLICT OF INTEREST

The authors state that they have no conflict of interest (COI).

## AUTHOR CONTRIBUTIONS

SM involved in concept, literature search, and drafting of manuscript. SY and MT involved in concept and literature search. SI‐Y involved in concept and revision of the manuscript.

## CONSENT

The written consent was obtained from the patient for the publication.

## Data Availability

The data that support the findings of this study are available from the corresponding author upon reasonable request.
